# Association between Blood Group and Nonmelanoma Skin Cancers (Basal Cell Carcinoma and Squamous Cell Carcinoma)

**DOI:** 10.3390/ijerph16132267

**Published:** 2019-06-27

**Authors:** Dijana Celić, Jasna Lipozenčić, Branko Kolarić, Goran Ferenčak, Jolanda Kanižaj Rajković, Tajana Borlinić

**Affiliations:** 1 Medikol Polyclinic, Franca Prešerna 13, 40 000 Čakovec, Croatia; 2 Croatian Academy of Medical Sciences, Praška 2, 10 000 Zagreb, Croatia; 3 Faculty of Medicine, University of Rijeka, Braće Branchetta 20/1, 51 000 Rijeka, Croatia; 4 Andrija Štampar Teaching Institute of Public Health, Mirogojska cesta 16, 10 000 Zagreb, Croatia; 5 Čakovec County Hospital, Ivana Gorana Kovačića 1E, 40 000 Čakovec, Croatia

**Keywords:** ABO blood groups, nonmelanoma skin cancers, basal cell carcinoma, squamous cell carcinoma

## Abstract

Background: Development of nonmelanoma skin cancers (NMSCs) has been associated with certain risk factors, but studies of the association between ABO blood group and NMSCs have been rare and inconclusive. The aim of this study was to assess the association of the previously known risk factors and blood group as a new potential risk factor in NMSCs. Methods: The study included 401 patients, 202 men, and 199 women, which included 367 diagnosed cases of basal cell carcinoma and 148 diagnosed cases of squamous cell carcinoma. The control group consisted of 438 subjects, 198 men, and 240 women. A standardized questionnaire adapted for this targeted study was used. The relation between the dependent variable (NMSCs) and independent variables was investigated by logistic regression. Results: Compared to the non AB blood group, the risk of developing NMSCs was significantly higher in the AB blood group (MOR = 2.28; 95% CI = 1.41–3.69). We established a logistic model that could best describe the probability of NMSCs development. Conclusion: Study results are expected to instigate basic research into the role of A and B antigens in normal skin epithelium, NMSCs etiopathogenesis, possible effect on metastatic potential and disease prognosis, potential tumor immunotherapy, and targeted detection and prevention in subjects at an increased risk of NMSCs development.

## 1. Introduction

Nonmelanoma skin cancers (NMSCs) is a term most frequently referring to basal cell carcinoma (BCC) and squamous cell carcinoma (SCC) [1]. These are the most common malignant tumors in Caucasians, with 80% and 20% incidence of BCC and SCC, respectively [1]. In spite of the growing awareness of the harmful effects of ultraviolet (UV) radiation as the major risk factor in the pathogenesis of these tumors, the incidence of NMSCs is on an increase in many countries, thus becoming an ever greater public health problem [2,3]. It is presumed to be influenced by a number of factors, such as damage to the ozone layer, which loses its protective role of filter for UV rays, prolonged life expectancy, earlier tumor detection through popularization of sun protection, the use of tanning salons, modified clothing, and an increasing number of organ transplantations. The latter is associated with long-term immunosuppressant therapy, which in turn entails the occurrence of malignant tumors, SCC being most common in this group of patients [2,3,4]. Generally, NMSCs are not regularly reported to national cancer registries; therefore, data on their incidence and mortality are not always complete. The incidence of NMSCs varies across the world, being highest in Australia (1000/100,000 person-years for BCC) and lowest in Africa (<1/100,000 person-years for BCC) [3]. Data on the incidence of NMSCs in Croatia are not published in annual reports of the National Cancer Registry because these tumors are believed to be under-reported. The only available data on the incidence of NMSCs are those for the 2003–2005 period [5,6,7]. The crude incidence rate of BCC was 54.9/100,000 for men and 53.9/100,000 for women, while the age standardized rates (as per standard world population) were 33.6/100,000 for men and 24.5/100,000 for women [5,6]. The crude incidence rate of SCC was 14.6/100,000 for men and 13.4/100,000 for women, whereas the age standardized rates (as per standard world population) were 8.9/100,000 for men and 5.2/100,000 for women [5,7].

Light skin phototype, some genodermatoses, exposure to ionizing radiation or chemical carcinogens, smoking, chronic inflammation and irritation, immunosuppression, as well as a history of NMSCs and melanoma increase the likelihood of NMSCs occurrence [8,9].

The association of blood groups and particular diseases is of great interest in medicine. The genetics and biochemistry of ABH antigens and their precursors expressed on red blood cells (RBC) is well known and described in detail [10]. However, the role of these antigens expressed on epithelial and endothelial cells, and in body excretions where they are normally found, as well as their role in the etiology of malignant tumors is by far less elucidated. In the majority of human carcinomas, a significant finding is decreased expression of A and B antigens on the cells of the carcinomas of the stomach, proximal colon, pancreas, larynx, lungs, endometrium, ovary, prostate, urinary bladder, kidney, breast and oral cavity [10,11,12,13,14].

Ghazizadeh et al. tried but failed to determine the role of the loss of A antigen expression on the penile SCC cell surface in relation to their concurrent expression on the surrounding healthy skin epithelium [15]. Other authors investigating expression of blood group antigens in carcinoma of the urinary bladder transitional epithelium made a step forward, confirming that noninvasive carcinoma (T1a stage) with ABH antigens expressed on their cells rarely invaded bladder wall, unlike the cases in which carcinoma cells did not express ABH antigens [16]. The loss of A and B antigen expression is caused by a reduced activity of ABO transferase in tumor cells in comparison to normal mucous epithelium [17,18,19]. The events preceding the loss of ABO transferase activity and function include loss of heterozygosity on chromosome 9 on which the locus for ABO gene is located, and in other cases, hypermethylation of the ABO promoter gene [20,21,22]. Considering that hypermethylation occurs exclusively on ABO locus but not on the neighboring genes, it has been concluded that hypermethylation is a tumor specific event [20,21,22]. Another possible mechanism beyond the ABO promoter is disorder of the ABO gene transcriptional regulation [10].

Blood group antigens have an important role in defining cell migration during both normal biologic processes and metastatic processes [22]. In the process of wound healing, epithelial cells lose A and B antigen expression. Upon completion of the healing process, expression of A and B antigens on epithelial cell membrane is resumed. Therefore, it has been concluded that A and B antigens prevent cell migration; accordingly, the loss of expression of these antigens on carcinoma cells would mean a higher metastatic potential, and thus poorer prognosis [22]. It is interesting to note that A and B antigens are also found on carcinoma cells in the tissues where they normally are not present [10]. Epithelial cells of fetal sigmoid intestine and rectum express ABO antigens, which are lost in adulthood, being retained only on the cells of proximal colon [23,24,25]. In cases of colorectal neoplasia, there is re-expression of A and B antigens on distal colon, supporting the theory on reactivation of the fetal oncogen function in analogy to the events associated with carcinoembryonic antigen (CEA) [23,24,25]. Highly dynamic blood group antigen expressivity is present in the human body, i.e., on both healthy epithelium and carcinoma cells. Therefore, it is quite disturbing how little we actually know about it, especially in cases where carcinoma cells may also express another blood group antigens, which is in contrast to their ABO RBC phenotype [10,26,27,28].

Review of the relevant literature points to the association of ABO blood groups and malignant diseases, with a predominance of the A blood group as compared with the O blood group [10].

The most consistent results refer to the association of the A blood group with gastric carcinoma and of non O blood groups with pancreatic carcinoma [10,29,30]. The association and role of A and B antigens in the genesis of NMSCs has not yet been fully clarified and the results published to date are not consistent [31,32,33].

The aim of the study was to assess the association of blood group and other potential risk factors with the development of NMSCs.

## 2. Materials and Methods

The study included 839 subjects treated at Dermatology and Venereology Clinic, Medikol Polyclinic in Čakovec (401 NMSCs patients and 438 patients as control group), aged 32–90, of Croatian origin, living in the Međimurje County for at least 20 years, as a convenient sample. Control group included subjects treated for other skin diseases. Exclusion criteria were as follows: subjects of origin other than the Republic of Croatia, patients with genodermatoses carrying a high risk of NMSCs (Gorlin syndrome, Bazex syndrome, Rombo syndrome, xeroderma pigmentosum, epidermodysplasia verruciformis, porokeratosis of Mibelli, and albinism), subjects with a history of exposure to ionizing (x-ray) radiation, immunodeficient states (state after organ transplantation or chemotherapy, immunosuppressive therapy, and HIV positive patients), melanoma patients, and subjects previously treated with phototherapy for other skin diseases.

Only standard diagnostic and therapeutic procedures were employed. Suspicion of skin tumor was set by clinical examination, and the diagnosis was confirmed by histopathology of the excision specimen. All study patients presented a written blood type report.

Using a questionnaire, detailed information was collected on demographic factors, personal history of any cancer, including skin cancer, family history of skin cancer, skin phototype, professional and recreational exposure to sunlight, history of sunburns before and after the age of 20 years, cigarette smoking and ABO blood type. A complete skin examination was performed by a dermatologist to assess pigmentary characteristics and the presence of sunlight induced skin lesions. All subjects gave their informed consent before they participated in the study. The study was conducted in line with ethical principles set in the Helsinki Declaration. The study protocol was approved by Ethics Committee of School of Medicine University of Zagreb and Ethics Committee of Medikol Polyclinic (identification code 04-76/2009-592). The strength of association between dependent (NMSCs) and independent variables was assessed using univariate logistic regression. To neutralize the potential effect of biological confounders we calculated odds ratios adjusted for age and sex. We also performed multiple logistic regression with all independent variables of interest. Finally, we developed a logistic model using a forward entry stepwise method. The selection criterion for variables to be included in the logistic model was *p* < 0.2 in univariate regression. Numerical variables were tested for normality of distribution after logistic transformation. The level of statistical significance was set at α = 0.05. The STATA/IC ver. 11.2 (StataCorp LLLC, College Station, TX, USA) software was used on data processing.

## 3. Results

In 401 study patients, 515 NMSCs were diagnosed, including 241 (46.8%) in women and 274 (53.2%) in men. Table 1 shows the NMSCs distribution according to sex, tumor localization, blood group and skin type.

Results of univariate and multiple logistic regression for NMSCs outcome are shown in Table 2.

A logistic model with the most significant NMSCs predictors is illustrated in Table 3.

## 4. Discussion

As early as 1945, E. B. Ford, a famous geneticist, wrote: “It is reasonable to conclude, from what we know of polymorphism, that individuals belonging to the different blood groups are not equally viable… A valuable line of enquiry which does not yet seem to have been pursued in any detail would be to study the blood group distributions in patients suffering from a wide variety of diseases. It is possible that in some conditions, infectious or otherwise, they would depart from their normal frequencies indicating that persons of a particular blood group are unduly susceptible to the disease in question” [34]. Gene frequency of the ABO blood type system varies among various populations, suggesting that a particular blood group prefers selection relative to the other blood groups, thus enabling survival in different ecologic conditions and exposure to various pathologic events [35]. Research into the association between blood groups and malignant tumors in various populations has opened way to studies at the molecular level trying to determine the role of acquiring and losing A and B antigen expression on carcinoma cells and their possible impact on the metastatic potential and disease prognosis. To our knowledge, such studies in NMSCs are almost completely lacking.

The ABO blood group A and B antigens are not only found on RBC membrane but also on the surface of many epithelial cells, including skin [10]. Ghazizadeh et al. report that in normal skin, A antigen is found in the granular layer of the epidermis, in the sebaceous and sudoriferous gland canals, and in the part of hair follicle root sheath passing through the granular layer [15]. Holborow et al. detected B antigen and H antigen type 2 chain in the granular layer, on cell membranes and in the cytoplasm [36]. In our study, we investigated the potential association between blood groups and development of NMSCs in a target population, which was the first study on this issue in Croatia. The study was limited to a single area in Croatia, Međimurje County, in order to avoid the effects of different geographical regions, primarily the effect of UV radiation, on study results.

Međimurje County is the northernmost county in Croatia with a population of 113 804 inhabitants, 55,601 male and 58,203 female, according to the last census from 2011 [37]. The effects of the known risk factors for this tumor type were also taken in consideration. The share of particular blood groups in the study subjects correlated with the blood group distribution in Croatia. Analysis of blood group distribution revealed the tumors to develop twice more frequently in the AB blood group.

Analysis of A, B, AB and non O (A, B, and AB) blood groups versus O blood group showed that only AB blood group is a significant predictor for NMSCs development by both univariate and multiple logistic regression. We assumed that the simultaneous presence of A and B antigens in the skin epithelium is possibly involved in the development of NMSCs. In accordance with the results we further analyzed the association of AB versus non AB blood group, which also showed the AB blood group to be a significant predictor by both univariate and multiple logistic regression analysis.

The effect of the known risk factors, UV radiation as the major one, on the occurrence of NMSCs was also assessed. The most common localization of NMSCs in the head and neck region can be explained by its chronic exposure to UV radiation, the association of which was also confirmed in our study. NMSCs localization on the trunk, upper and lower extremities can be considered consequential to intermittent exposure to UV radiation, as also reported elsewhere [5,6,7,8,9]. Therefore, protection from UV radiation is emphasized as a measure of NMSCs prevention. Lesions occurring due to UV radiation accumulate throughout a lifetime, thus older age carries a higher risk of NMSCs, as demonstrated in our study.

Men are involved more frequently than women because they are more likely to work outdoors. However, literature data reveal that these sex differences are on a steady decrease [5,6,7]. In our study, multiple regression analysis did not confirm the association of sex and NMSCs, probably because women from the study population were also engaged in agriculture and gardening, which implies intermittent sun exposure, along with free time spent outdoors. Another possible explanation is the fact that, according to the last census from 2011, there are more women than men aged 60–95+ (37). The age median in our study patients was 68, range 32–90 years, which could explain the higher rate of NMSCs in women.

Occupational exposure to UV radiation is variedly defined in the literature but most authors define it as more than 3-h/day exposure to UV radiation [38]. Our study results did not confirm the association of NMSCs with occupational exposure to UV radiation in multiple logistic regression. Significant result was association of NMSCs and leisure time spent outdoors versus indoors; in our study population, the latter should be interpreted as chronic exposure to UV radiation because they spent their free time in agriculture/gardening. Agricultural activities are intensive over seven months, from April to October, so it should be considered as occupational exposure to UV radiation. Living in rural areas is therefore linked to occupational exposure to UV radiation, and possibly also to a lower level of information and awareness of the adverse effects and consequences of sunburns. In our study, multiple regression analysis showed the result on the time equally spent indoors and outdoors versus indoors to be significant; it could be attributed to the intermittent exposure of our study population to UV radiation, which increases the likelihood of NMSCs development.

Childhood is the most sensitive life period that correlates with exposure to UV radiation and the occurrence of NMSCs later in life. Our study results pointed to sunburns acquired before and after the age of 20 years to be a significant predictor of NMSCs, but their association was lost in multiple regression analysis, indicating the need of photoprotection as a significant measure of NMSCs prevention throughout life irrespective of the age in which solar lesions occurred. Light skin individuals that always develop sunburns but never develop skin pigmentation are most sensitive to the action of UV radiation. According to Fitzpatrick (FP), skin sensitivity to UV radiation is categorized into six types, from very light skin (FP I-II) to very dark skin (FP VI) [39]. This categorization is determined by genetic characteristics, which are constant and unchanging, and refer to the amount of melanin in melanocytes, which influences skin color and sensitivity to UV radiation [39]. Skin types I and II are most commonly affected by NMSCs, but it is not rare in FP III type too [8,9]. In our study, FP II skin type was found to be a significant isolated predictor of NMSCs as compared with FP III and IV skin types. However, when multiple regression analysis was conducted, this association of NMSCs and FP II versus FP IV skin type was lost. The possible explanation for this is the low number of tumors in FP IV skin type. The presence of solar lentigo, actinic keratoses and actinic elastosis as the symptoms of solar skin lesions in NMSCs patients is often discussed in epidemiologic studies. In our studies, these entities were significantly associated with the occurrence of NMSCs. Therefore, their clinical recognition is of great importance as a measure of prevention and early detection of NMSCs in primary healthcare, where physicians should regularly inform and remind patients on the need of consistent protection from UV radiation and sunburns, with periodical patient referral to dermatologic examination. Familial occurrence of NMSCs has been described in some genodermatoses [8,9], which were an exclusion criterion in our study. That is why their association with NMSCs was not assessed. According to the current state-of-the-art on the issue, smoking can be one of the risk factors for NMSCs [40]. However, it was not confirmed in our study. This could be explained by the fact that the questionnaire used did not contain specific data on the number of cigarettes per day and the length of smoking habit. There is an open question of screening for skin carcinoma in smokers who more frequently develop malignant diseases of the respiratory, gastrointestinal and circulatory systems, which in turn may result in lethal outcome in young and middle age, i.e., before developing NMSCs. Accordingly, such a scenario may have masked the association of smoking and NMSCs in our patients.

Finally, we proposed a logistic model emphasizing the predictive value of those variables that describe best the likelihood of NMSCs development. Our results indicated a significant association of AB blood type and NMSCs. In comparison to non AB blood group, the AB blood group increased the likelihood of NMSCs 2.36 times in older age; in FP II versus FP III skin type; in the skin with initial solar skin lesions (sunburns) before age 20; in the skin at intermittent or chronic exposure to UV radiation; in the skin with actinic keratoses, actinic elastosis and solar lentigines, as a specific and objective symptoms of solar skin lesions.

It is presumed that the concurrent presence of A and B antigens in the skin epithelium increases the probability of NMSCs development. The association of AB phenotype and NMSCs recorded in our study could be explained by the greater resistance to apoptosis of epithelial cells expressing A or B antigens as compared with the cells that express exclusively H antigens [41]. UV radiation as a known factor that increases the likelihood of NMSCs, as also confirmed in our study, may act as a trigger for carcinogenesis, while the increased resistance to apoptosis of the cells expressing both A and B antigens may be responsible for promotion and progression of carcinogenesis. The cells expressing A and B antigens will therefore be more resistant to apoptosis, thus being more difficult to eliminate in case of adverse mutations during tumorigenesis. The carcinoma cells that express A antigen are also characterized by a considerably higher resistance to apoptosis induced by heat shock proteins [42], which could additionally contribute to the progression of carcinogenesis.

Tursen et al. did not confirm the association of the ABO blood group and NMSCs [31]. Cihan et al. monitored blood group distribution and differences between NMSCs patients and control group, suggesting additional research focused on the A Rh- blood group [32]. Results reported by Xie et al. reveal that non O blood groups has a lower likelihood of developing SCC and BCC than the O blood group; their study included Caucasians, however, without data on their origin, which is important information considering the ethnic heterogeneity of the USA population and the fact that the frequency of human genes of the ABO system varies among different populations and ethnic groups [10,33]. The authors explain the lower likelihood of NMSCs in individuals with the A blood group by lower expression of A antigen in penile SCC relative to the surrounding normal skin (15). Carcinoma cells are known to lose A and B antigen expression as a consequence of decreased ABO transferase activity in tumor cells in comparison to normal epithelium [10,17,21].

Consistent to our results on the association of NMSCs and the AB blood group versus non AB blood groups, other authors also confirmed the association of AB blood group and particular types of carcinoma. The rate of the AB blood group is the lowest worldwide. It is most commonly found in Japan and in some parts of China and Korea (up to 10% of the population) [43]. A study conducted in the population of southeast China demonstrated the association of nasopharyngeal carcinoma with the A blood group and the AB blood group versus the O blood group (AOR = 1.287; 95% CI = 1.072–1.545 and AOR = 1.390; 95% CI = 1.007–1.919, respectively) [44]. A study by Ben et al. in a Chinese population confirmed the association of pancreatic carcinoma with the A blood group and the AB blood group versus the O blood group (AOR = 1.368; 95% CI = 1.127–1.661 and AOR = 1.391; 95% CI = 1.053–1.838, respectively) [45]. A study conducted in Taiwan confirmed the association of all carcinomas (lungs, liver, pancreas, esophagus, stomach, colon and prostate) in men with AB blood group as compared with the O blood group (HR = 1.66; 95% CI = 1.20–2.30) [46]. Analysis of the association of particular carcinomas and blood groups in men confirmed the association of the AB blood group versus the O blood group with carcinoma of the lungs and gastrointestinal system (HR = 2.64; 95% CI = 1.31–5.33 and HR = 1.86; 95% CI = 1.16–2.97, respectively) [46]. In addition, this study confirmed the association of all the above-mentioned carcinomas in both men and women with A or A/AB antigens versus O/B antigens (men: HR = 1.20; 95% CI = 1.01–1.42; women: HR = 1.29; 95% CI = 1.07–1.56) [46]. In women, breast and cervical cancers were also included [46].

The limitations of our study refer to particular data that were based on patient recall and self-assessment, i.e., data on sunburns sustained before and after age 20. Some study subjects may have omitted such data, being unwilling to report something that is currently considered as a lack of care for one’s health. Intensive actions for protection from UV radiation have been launched through the widely available mass media. However, in our patients, the effect of UV radiation was manifested by specific and objective skin symptoms verified by clinical examination (solar lentigines, actinic keratoses and actinic elastosis). Another potential limitation was the lack of reliable data on smoking habits. Some chronic smokers may have quit smoking several months before and marked themselves as nonsmokers in the questionnaire. This question did not include data on the length of smoking time and number of cigarettes per day.

## 5. Conclusions

Study results demonstrated the AB blood group and A and B antigens to be predictive of carcinogenesis, as they were significantly associated with the occurrence of NMSCs in our study population.

Our results pointed to the need of additional basic research into the role of A and B antigens in normal skin epithelium, NMSCs etiopathogenesis, possible effect on metastatic potential and disease prognosis, potential tumor immunotherapy, and targeted detection and prevention in subjects at an increased risk of NMSCs development.

## Figures and Tables

**Table 1:** Distribution of nonmelanoma skin cancers (NMSCs).

		Control Group, n (%)	NMSCs Group, n (%)
Sex	Female	240 (54.8)	241 (46.8)
	Male	198 (45.2)	274 (53.2)
Tumor localization	Head and neck		338 (65.6)
	Trunk		122 (23.7)
	Arms		47 (9.1)
	Legs		8 (1.6)
Blood group	A	171 (39)	196 (38.1)
	B	77 (17.6)	89 (17.3)
	AB	33 (7.5)	70 (13.6)
	O	157 (35.8)	160 (31.1)
Skin type	I	0 (0)	6 (1.1)
	II	104 (23.7)	228 (44.2)
	III	320 (73.1)	268 (52.0)
	IV	14 (3.2)	13 (2.5)

**Table 2:** Results of logistic regression for nonmelanoma skin cancers (NMSCs) outcomes.

Characteristic	OR (95% CI)	AOR (95% CI)	MOR (95% CI)
Sex (male vs. female)	1.36 * (1.06–1.78)	NA	1.17 (0.86–1.58)
Age (yrs)	1.05 * (1.03–1.06)	NA	1.02 * (1.01–1.04)
Occupation (outdoors vs. indoors)	2.1 * (1.62–2.74)	1.61 * (1.22–2.13)	1.21 (0.88–1.67)
Blood group (AB vs. non AB)	1.93 * (1.25–2.98)	1.96 * (1.25–3.08)	2.28 * (1.41–3.69)
Cigarette smoking	0.96 (0.73–1.28)	1.15 (0.85–1.57)	1.25 (0.9–1.74)
Skin type II vs. III	2.63 * (1.96–3.44)	2.5 * (1.85–3.33)	1.96 * (1.40–2.72)
Skin type II vs. IV	2.38 * (1.07–5.26)	2.04 (0.95–4.76)	1.29 (0.55–3.03)
Solar lentigo	2.03 * (1.49–2.76)	1.5 * (1.07–2.09)	1.66 * (1.15–2.39)
Actinic keratosis	5.02 * (3.5–7.19)	3.66 * (2.49–5.37)	2.34 * (1.52–3.58)
Actinic elastosis	3.63 * (2.73–4.83)	2.7 * (1.98–3.67)	1.64 * (1.15–2.36)
Positive family history	1.94 * (1.17–3.2)	2.07 * (1.23–3.46)	1.62 (0.93–2.84)
Free time spent indoors and outdoors vs. indoors	1.46 (0.94–2.26)	1.6 * (1.02–2.53)	1.64 * (1.01–2.66)
Free time spent outdoors vs. indoors	2.42 * (1.55–3.76)	2.31 * (1.46–3.66)	1.73 * (1.05–2.85)
Sunburns before age 20	1.67 * (1.29–2.16)	1.65 * (1.26–2.16)	1.27 (0.93–1.75)
Sunburns after age 20	1.75 * (1.35–2.27)	1.6 * (1.22–2.09)	1.22 (0.89–1.67)

* statistically significant at the level α = 0.05; OR, univariate logistic regression odds ratio; 95% CI, 95% confidence interval; AOR, odds ratio adjusted for age and sex; MOR, multiple logistic regression odds ratio; NA, not applicable.

**Table 3:** Logistic model for nonmelanoma skin cancers (NMSCs).

Characteristic	MORm	95%CI (OR)
Sex (male vs. female)	1.23	0.92–1.66
Age (yrs)	1.02 *	1.01–1.03
Blood group (AB vs. *non* AB)	2.36 *	1.47–3.8
Skin type II vs. III	2.0 *	1.44–2.72
Skin type II vs. IV	1.35	0.58–3.12
Solar lentigo	1.64 *	1.15–2.35
Actinic keratoses	2.32 *	1.52–3.54
Actinic elastosis	1.72 *	1.21–2.44
Free time spent indoors and outdoors vs. indoors	1.65 *	1.02–2.67
Free time spent outdoors vs. indoors	1.88 *	1.15–3.07
Sunburns before age 20	1.12 *	1.06–1.9

* statistically significant at the level α = 0.05; 95% CI, 95% confidence interval; MORm, odds ratio in multiple logistic model.
